# Compound Kushen Injection for gastric cancer: A protocol of systematic review and meta-analysis

**DOI:** 10.1097/MD.0000000000017927

**Published:** 2019-11-11

**Authors:** Zhihong Huang, Ping Wei

**Affiliations:** Donggang Branch of The First Hospital of Lanzhou University, Lanzhou, China.

**Keywords:** Compound Kushen Injection, efficacy, gastric cancer, meta–analysis, safety, systematic review

## Abstract

**Background and aims:**

In recent years, the clinical research about Compound Kushen Injection (CKI) treatment of Gastric cancer (GC) has been increased, but the conclusion is different. The aim of our study is to objective comment the efficacy and adverse effects of CKI treatment of GC.

**Methods:**

We will retrieve the Randomized controlled trials from the following 6 electronic databases on their inception to April 2019: PubMed, Embase, Cochrane Library, China National Knowledge Infrastructure, Wangfang and Chinese Biomedical Literature Database. Study selection and data collection will be performed independently by 2 reviewers. Cochrane Risk of Bias tool will be used to assess the risk of bias of included studies. The outcomes included overall response rate, complete response rate, 3–year progression–free survival rate, 3–year overall survival rate, and different types of treatment–related adverse events. We calculated the risk ratios as well as their 95% confidence intervals of these outcomes and pooled the results using RevMan 5.2 software and Stata 14.0 software.

**Results:**

The results will be published in a peer-reviewed journal.

**Conclusion:**

The results of this review will be widely disseminated through peer-reviewed publications and conference presentations. This evidence may also provide helpful evidence for clinical practice and health policy-makers for the treatment of GC.

**PROSPERO registration number:**

CRD42019133770.

## 1 Introduction

Gastric cancer (GC) is a multifactorial disease in which both environmental and genetic factors play a role in its etiology. It has a common high-fatality disease that approximately 990,000 people are diagnosed with GC worldwide each year.^[1]^ The prognosis is dismal because of late diagnosis, with an average 5-year survival rate of less than 20%.^[1]^ It is the second leading cause of cancer death after lung cancer, especially in Eastern Asia (such as China, Japan et al).^[2]^ GC causes the highest cancer burdens, whether in developed or developing countries.^[3]^ Measures such as cigarette control and eradication of Helicobacter pylori reduced the incidence of GC worldwide over the last 50 year.^[4]^ However, other factors such unhealthy diet, obesity, increasing work pressure et al, will affect the incidence of GC in future.

Reducing the mortality of GC in addition to early prevention, treatment is also very important. For GC, different clinical stage treatments are different, such as endoscopic submucosal dissection, local tumor resection, neoadjuvant chemotherapy, adjuvant chemotherapy, adjuvant chemoradiotherapy, et al.^[5–8]^ At present, there are differences between East and West scholars on the causes of different survival rates in the East and West of gastric cancer.^[9,10]^ In general, the treatment of gastric cancer in the East and West is not exactly the same. Some scholars even have called for Bridge the West and the East in the Treatment for GC.^[11]^

In addition to focusing on the survival rate of cancer, how to improve the quality of life of cancer patients has also become important. Traditional Chinese interventions have huge advantages in treating cancer and improving the quality of life of patients. The nonpharmacologic interventions include a series of approaches such as acupoint stimulation, Chinese massage (referred to as “Tuina”), Tai Chi, Qigong, and Traditional Chinese Medicine Five-Element Music Therapy.^[12]^ The main pharmacologic interventions are traditional soups and proprietary Chinese medicines (main including oral and injectable).^[13–15]^ Compound Kushen Injection (CKI) has been used in combination with chemotherapies for the treatment of cancer (such as GC, liver and non-small cell lung carcinomas) since 1995.^[16]^ It is now being used for the treatment of more types of cancer.^[17]^ At present, CKI can cure cancer because it can boost immunity, decrease inflammation, and decrease metastasis.^[18]^ In recent years, the clinical research about CKI treatment of GC has been increased, but the conclusion is different.^[19,20]^ Some scholars have summarized the efficacy and adverse effects (AEs) of treating advanced GC.^[21]^ We are prepared to summarize the efficacy and AEs of CKI treatment of GC at any stages through the meta-analysis (MAs).

## 2 Study aim

The aim of our study is to objective comment the efficacy and AEs of CKI treatment of GC. A better understanding of CKI, guide the clinical treatment of GC patients.

## 3 Methods

The protocol of our MAs followed the guideline of the Preferred Reporting Items for Systematic Review and Meta-Analysis Protocols (PRISMA-P) recommendations.^[22]^ The protocol has been registered in Prospective Register of Systematic Reviews, with the registration number: CRD42019133770.^[23]^ If we need to change this protocol, the changes would be described in our full review and will be tracked in the International Prospective Register of Systematic Reviews.

### 3.1 Eligibility criteria

#### 3.1.1 Types of studies

Randomized controlled trials (RCTs) of CKJ for GC, irrespective of blinding, publication status and language, will be included to pool and review in this study. Non-RCTs, observational studies, qualitative studies, and laboratory studies et al will be excluded.

#### 3.1.2 Types of participants and interventions

Trials included adult (18 years or older) participants of any ethnic origin, gender, nationality who had GC (The diagnostic criteria will be developed according to the International Classification). Patients with other malignancies or non-primary GC are not included. As long as the trials reflect CKI intervention, regardless of whether or not other treatments are accepted. It is to say, interventions to be reviewed are CKI alone or combinations with other interventions to treat the GC. When CKI used as combinations with other treatments, the control group should also receive the same combination treatments.

#### 3.1.3 Types of outcome

Outcomes of our MAs will include overall response (OR), complete response (CR), 3–year OS, 3–year progression-free survival (PFS) and treatment–related AEs. OS is defined as the time from the date of randomization to death from any cause. PFS is defined as the time from the date of randomization until disease progression or death. As for AEs, we will analyst the Grade 3 or 4 blood and non-hepatotoxicity.^[24]^

### 3.2 Search strategy

The following databases including 3 English medical databases and 3 Chinese databases will be searched from their inception to April 2019: PubMed, Embase, Cochrane Library, China National Knowledge Infrastructure (CNKI), Wangfang and Chinese Biomedical Literature Database. The MeSH search and text word will be used with the terms related to GC and CKI. To perform a comprehensive and focused search, experienced systematic review researchers will be invited to develop a search strategy. The plan searched terms are as follows: gastric cancers, stomach neoplasms, gastric carcinomas and Fufangkushen, Compound Kushen Injection et al. An example of search strategy for PubMed database shown in Table 1 will be modified and used for the other databases. The reference lists of all relevant studies will be searched for additional relevant studies not retrieved from the electronic database search.

**Table 1:**
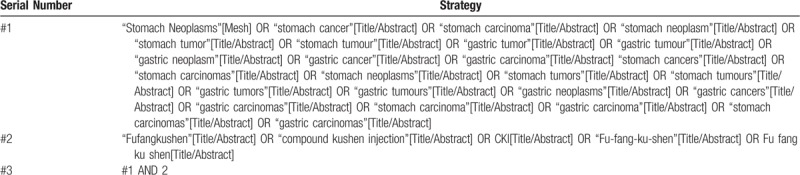
Searching strategy in PubMed.

### 3.3 Study selection

**Figure 1:**
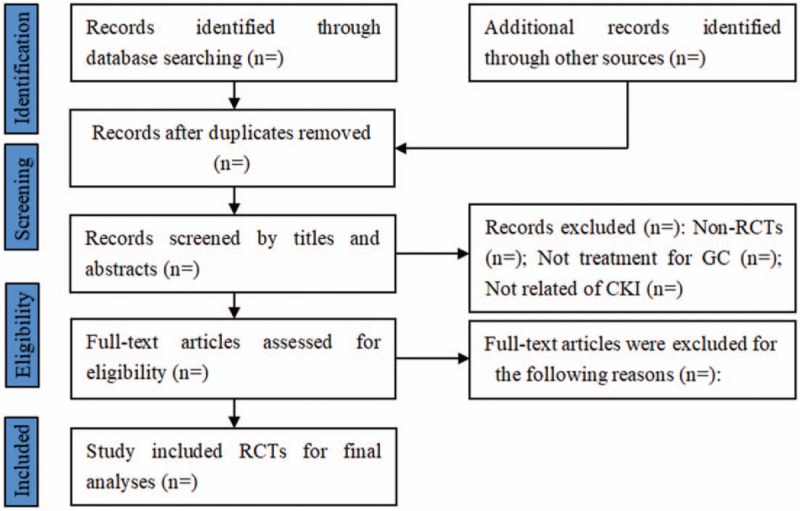
Study selection for meta-analysis. GC = gastric cancer, RCTs = randomized controlled trials.

All initial records from 6 electronic databases will be imported into the web-based systematic review software package “Rayyan”.^[25]^ First, the titles and abstracts of records will be reviewed independently by 2 reviewers to identify potential trials according to eligibility criteria. Then, full-text of all potentially relevant trials will be downloaded to make sure eligible trials. Any conflict will be resolved by discussion. A flow diagram (Fig. 1) will be used to describe the selection process of eligible papers.

### 3.4 Data extraction and management

The data was extracted out by 2 independent reviewers in accordance with the standardized sheet recommended by the Cochrane Handbook of Systematic Reviews of Interventions. The following data will be extracted from eligible studies using pilot-tested data extraction forms: author, year of publication, country where the study was performed, funding, study duration, contact details of the authors, sequence generation, allocation sequence concealment, blinding, incomplete outcome data, and selective outcome reporting, participants (total number, setting, age, sex, country, diagnostic criteria for GC, etc.), details of all experimental and control interventions (manufacturer of the drugs, dosages, medication route, duration of treatment, etc.), outcomes (OR, CR, OS, PFS, AEs). Important missing data were obtained by contacting article authors whenever possible. Data extraction will be completed independently by paired reviewers. Any conflicts will be resolved by discussion.

### 3.5 Risk of bias of individual study and quality assessment

Two reviewers will evaluate independently the risk of bias of included RCTs using a modified version of Cochrane tool,^[26]^ in which we will to check for random sequence generation, allocation concealment, blinding, incomplete outcome data, selective reporting, and other bias, each of which makes high-risk, low-risk, and unclear grades.

We will use the Grading of Recommendations Assessment Development and Evaluation (GRADE) system to assess the quality of the evidence by 2 reviewers.^[27]^ Disagreements between the 2 reviewers will be resolved by a third reviewer during the evaluation of bias and quality assessment.

### 3.6 Data synthesis

Statistical analyses will be performed using Review Manager 5.3 statistical software (Cochrane Collaboration, Denmark) and Stata 14.0 software (Stata Statistical Software: Release 14. College Station, TX: StataCorp LP). The outcomes will be presented as the relative risk, mean difference or standardized mean difference and its 95% confidence interval (95% confidence intervals (CI)). The statistical significance will be assessed for *P* < .05, and moderate to high levels of heterogeneity will be considered for *I*^2^ > 50%.^[27]^ A fixed effects model will be used if no statistical heterogeneity across the studies; otherwise, the random effects model will be considered.

### 3.7 Publication bias

If ≥10 included studies are available, funnel plot will be used to identify the possible publication bias. Additionally, Egg regression and Begger tests will be utilized to detect the funnel plot asymmetry.^[28]^

### 3.8 Subgroup analysis

If there is enough research, we will conduct a subgroup analysis to investigate differences in age, dose, and tumor stage et al.

## 4 Discussion

Traditional Chinese medicine injection has been widely used in cancer treatment, and its efficacy has been confirmed. This systematic review may provide a detailed summary of current evidence of CKI treatment GC. The results of this review will be widely disseminated through peer-reviewed publications and conference presentations. This evidence may also provide helpful evidence for clinical practice and health policy-makers for the treatment of GC.

## Author contributions

Conceptualization: Zhihong Huang, Ping Wei Funding acquisition: Zhihong Huang, Ping Wei Methodology: Zhihong Huang, Ping Wei Project administration: Zhihong Huang, Ping Wei Writing – original draft: Zhihong Huang, Ping Wei Writing – review & editing: Zhihong Huang, Ping Wei.
